# Cytometric evaluation of intracellular IFN-γ and IL-4 levels in thyroid follicular cells from patients with autoimmune thyroid diseases

**DOI:** 10.1186/1756-6614-4-13

**Published:** 2011-09-23

**Authors:** Artur Bossowski, Jerzy Harasymczuk, Anna Moniuszko, Anna Bossowska, Maciej Hilczer, Karol Ratomski

**Affiliations:** 1 Department of Pediatrics, Endocrinology, Diabetology with the Cardiology Division, Medical University. Bialystok 15-089. Poland; 2 Department of Pediatric Surgery, Traumatology and Urology. University of Medical Sciences, Poznań 60-967. Poland; 3 Department of Infectious Diseases and Neuroinfections. Medical University. Białystok 15-089. Poland; 4 Division of Cardiology. Internal Affair and Administration Ministry Hospital. Białystok 15-089. Poland; 5 Department of Endocrinology and Metabolic Diseases. Medical University. Łódź 93-338. Poland; 6 Dep. of Pediatric Laboratory Diagnostic. Medical University. Bialystok 15-089. Poland

**Keywords:** thyrocytes, cytokines, Graves, disease, Hashimoto's thyroiditis

## Abstract

**Background:**

In recent few years is underlined that altered balance of pro- and anti-inflammatory cytokines play an important role in the pathogenesis of AITD.

The aim of this study was to estimate intracellular INF-γ and IL-4 levels in thyroid-infiltrating lymphocytes and thyrocytes isolated from thyroid tissues in 54 adolescent patients aged 8-21 years, with Graves' disease (GD; n = 18), Hashimoto's thyroiditis (HT; n = 18) and non-toxic multinodular goiter (NTMG; n = 18).

**Methods:**

Fresh thyroid tissues were taken on culture medium RPMI -1640, it was mechanically prepared. In next step were added cell activators -12- myristate 13- the acetate (PMA) and Ionomycin as well as the inhibitor of transportation of proteins - Breferdin A. They were cultured 24 hours in 50 ml flasks at 37°C in a 5-95% CO2-air water-saturated atmosphere. After that, thyrocytes were identified by mouse mAb directed against human TPO epitope 64 conjugated with rabbit anti-mouse antibodies IgG (Fab')_2 _labeled by FITC. After incubation at room temperature to each of samples added reagent A fixative the cellular membrane. In next step into the cell suspensions were added reagent B to permeabilization of cellular membrane and specific anti-IL-4-PE or anti-IFN-γ-PE mAbs. Identification of intracellular cytokines in T lymphocytes was performed in the same procedure with application of anti-CD4-PerCP and anti-CD8-PerCP mAbs specific for T lymphocytes. The cells were analyzed in a flow cytometry (Coulter EPICS XL).

**Results:**

In examined group of patients with GD we observed statistically significant higher mean percentage of cells with phenotype CD4+IL-4 (p < 0.05; p < 0.025), CD8+IL-4 (p < 0.033; p < 0.01) and TFCs-IL-4+ (p < 0.05; p < 0.01) in comparison to patients with HT and NTMG. The analysis of mean percentages of positive TILs and TFCs with intracellular INF-g levels in patients with HT revealed statistically significant increase percentage of CD4+INF-γ (p < 0.04; p < 0.001), CD8+ INF-γ (NS; p < 0.025), TFCs+INF-γ (p < 0.03; p < 0.001) cells in comparison to the percentage of positive cells from patients with GD and NTMG.

**Conclusions:**

We conclude that human thyrocytes in autoimmune thyroid disorders could be a source of cytokine production and that their activation influences local interaction with T lymphocytes inflowing to the thyroid gland.

## Background

Graves' disease (GD) and Hashimoto's thyroiditis (HT) are a common autoimmune disorders. It was proven, that subpopulation of Th1 plays an important role in induction of classical mechanisms of late type reaction and that produced by CD4+ IFN-γ is an activator of macrophages and stimulates production of IgG2a. Th1 cells through the influencing on differentiation of cytotoxic lymphocytes CD8+, modulate also inflammatory reaction in response to the antigen stimulation [1]. CD4+ cells, which belong to subpopulation Th2 stimulate immunological response of B lymphocytes. There are some studies evaluating cytokines in these diseases, demonstrating the production of IL-4 and TNF- by infiltrating T cells and macrophages. However, the specific role of these molecules in the pathogenesis of autoimmune thyroid diseases (AITD) is still debated [2-4]. We presume that an altered balance of pro- and anti-inflammatory cytokines may play an important role in the pathogenesis of autoimmune thyroiditis.

T-helper 1 (Th1) cell-mediated inflammatory responses predominate in the early pathogenesis of GD, whereas Th2 cell-mediated immunity may play a role in later stages. Th1 cells produce IFN-gamma and Th2 cells produce IL-4. Nanba T et al. reported that IFN-gamma and IL-4 gene polymorphisms, which are related to higher IFN-gamma and lower IL-4 production, respectively, are more frequent in patients with severe HT than in those mild HT. They investigated the proportion of peripheral Th1 and Th2 cells in patients with AITD and concluded that the peripheral Th1/Th2 cell ratio is related to the severity of HT and is related to the intractability of GD. They hypothesize that these patterns of peripheral Th cell subsets may be expressed within the thyroid [5].

The aim of this study was to estimate intracellular INF-γ and IL-4 levels in thyroid-infiltrating lymphocytes (TILs), and in thyroid follicular cells with application of mouse monoclonal antibodies type #64 which recognize B antigen regions of TPO (thyroid peroxidase) in patients with AITD and NTMG.

## Patients and methods

The study was performed in a group of 54 adolescent patients (14 boys and 42 girls), aged 8-21 years, with Graves' disease (GD; n = 18, mean age 16.4 ± 3.3 years), Hashimoto's thyroiditis (HT; n = 18, mean age 16.8 ± 2.8 years) and non-toxic multinodular goiter (n = 18, mean age 16 ± 3 years), hospitalized in the Department Pediatrics, Endocrinology, Diabetology with the Cardiology Division, Medical University of Białystok. The patients underwent total or subtotal thyroidectomy in the 1st Department of General Surgery, Medical University of Białystok or in the Department of Pediatric Surgery, Traumatology and Urology, Poznań University of Medical Sciences. The diagnosis was established based on clinical examinations confirmed by laboratory, ultrasonographic and scintigraphic investigations with the use of ^131^I (in case of nodular goiter with symptoms of hyperthyroidism). Additionally, fine-needle aspiration biopsies in HT and nodular goiter were performed in the Department of Patomorphology, Medical University of Białystok. The qualifying criteria for patients with GD were as follows: large goiter, presence of ophthalmopathy, antibodies against receptor for thyroid stimulating hormone (TRAb) > 5, positive titers of antithyroid peroxidase (anti-TPO) or anti-thyroglobulin (anti-TG) antibodies, and persisting over 2-3 months since the diagnosis of thyroid- stimulating hormone (TSH) < 0.45. Methimazole therapy, at the initial dose of 0.5-1.0 mg/kg/day, was used in combination with propranolol 0.5-1.0 mg/kg/day to treat hyperthyroidism in the course of GD. A further reduction in methimazole dose and obtaining euthyrosis prior to surgery depended on clinical-biochemical parameters. Average daily doses of this antithyroid drug were 10-15 mg. Patients with HT received treatment exclusively with L-thyroxine (mean 75 ± 25 mg/24 h) for the period of 6-12 months (mean 8 months) since the diagnosis.

The function of the thyroid gland in patients with GD was assessed at the time of diagnosis and prior to surgery. Thyroid function was evaluated based on thyroid hormones and TSH tests performed jointly with the measurement of titers of antithyroid antibodies (ATPO, ATG, TRAK).

Our study was approved by the Committee for Ethics and Supervision on Human and Animal Research of the Medical University in Białystok. An informed consent was given by the participating children and their parents.

### Determination of the Antithyroid Antibody Titers and Thyroid Hormone Concentration

Blood for analysis was collected on empty stomach in the morning hours from the basilic vein and centrifuged for 10 min at 2,000 rotations/min. Sera were stored at -20° until the required number was collected. Thyroid stimulating hormone receptor antibodies (TRAbs) were routinely determined by a radioreceptor assay (TRAK-human kits, Brahms Diagnostica GmbH, Berlin, Germany), in which the value < 1 U/l was treated as negative, 1.0-1.5 U/l as a grey zone and the values > 1.5 U/l were considered positive. Antithyroperoxidase antibodies (TPO-Abs) and antithyroglobulin antibodies (TG-Abs) were determined using immunodiagnostic test Varelisa (Variable Enzyme Linked Immno Sorbent Assay, Pharmacia

Upjohn Diagnostics GmbH Co.KG. Freiburg, Germany). Normal range for TG-Abs was < 500 IU/ml and for TPO-Abs was < 50 IU/ml. The levels of fT4, fT3 and hTSH in blood serum were determined on a mini-analyzer (Bio Merieux, France), based on test VIDAS (Bio Merieux, France), combining the immunoenzymatic method with the final fluorescence measurement (ELFA). Normal range of fT4 was 0.71-1.55 ng/dl, fT3--2.6-5.4 ng/dl and hTSH--0.32-5.0 μIU/ml.

### Thyrocyte preparations

The thyroid tissue samples were analyzed following the previously described techniques [6,7]. Briefly, postoperative 1.0 cm/1.0 cm thyroid sections were collected to test-tubes with RPMI-1640 medium. Initially, the material was minced mechanically and digested with a mixture of collagenase type IV-S (Sigma) in Hanks balanced saline solution (HESS). To eliminate non-adherent cells from thyrocyte preparations, the dispersed cells were cultured for

**Figure 1:**
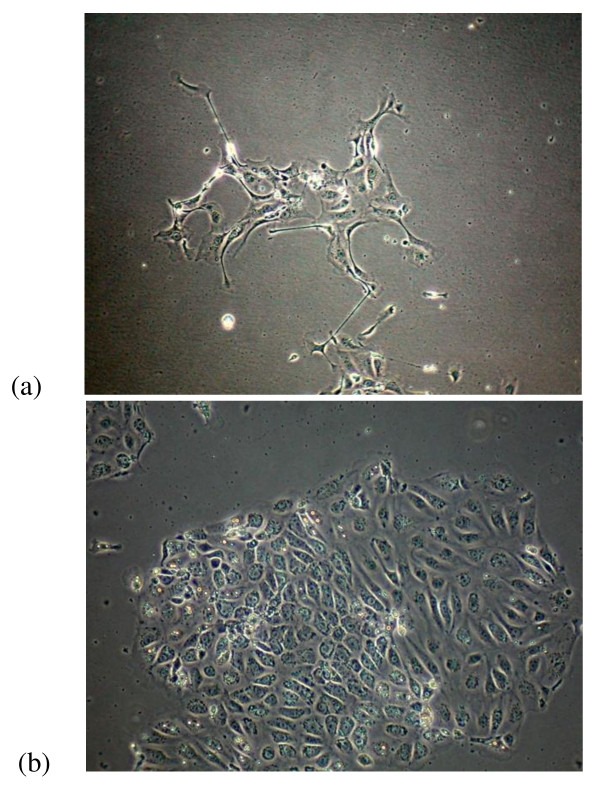
**Thyrocytes in suspension in culture medium (a) formed monolayers on plastic plates after 24 h in culture (b)**.

24 h with minimum essential medium (MEM), containing 10% fetal bovine serum and Ham F12 medium, supplemented with 20 mm HEPES buffer, 2 mm L-glutamine, 100 U/ml penicillin, and 100 μg/ml streptomycin (Sigma). They were cultured in 50 ml flasks (Falcon Plastics, Becton-Dickinson, USA) at 37°C in a 5-95% CO2-air water-saturated atmosphere. The following day, the non-adherent cells (predominantly lymphoid cells and erythrocytes) were removed from culture supernatants for lymphocyte isolation. Trypsinization was performed at confluence (Figure 1).

### Identification of thyrocyte populations

Purity of thyroid cells was tested by flow cytometry as previously described [7]. Briefly, the isolated cells diluted in RPMI 1640 medium were rinsed twice with a buffer containing PBS, 0.5% bovine albumin and 2 mM EDTA, and suspended in 1 ml PBS. After that, 50 μl portions of cell suspension were placed in a test-tube, adding 10 μl of mouse monoclonal antibody (mAb) at 400 μg/ml concentration directed against human TPO epitope 64 reacting with domain B. Following 30 min incubation at 4°C, the cells were rinsed three times with a buffer of PBS + 0.5% bovine albumin + 2 mM EDTA. Then, 20 μl of rabbit antibody F(ab')_2 _conjugated with FITC (FITC-coupled F(ab')_2 _antimouse IgG, DAKO) was added. After 30 min incubation at 4°C, cell suspensions were rinsed three times again in the buffer described above. Then, the samples were submitted to analysis in a flow cytometer (Coulter EPICS XL).

The unspecific binding of monoclonal antibodies to the thyrocytes was evaluated by the use of irrelevant IgG of the same isotype as the used antibodies. The specificity of the reaction was checked by immunoprecipitation of mAbs by highly purified human TPO.

### Identification of intracellular IL-4 and IFN-γ levels in thyrocytes by flow cytometry

**Figure 2:**
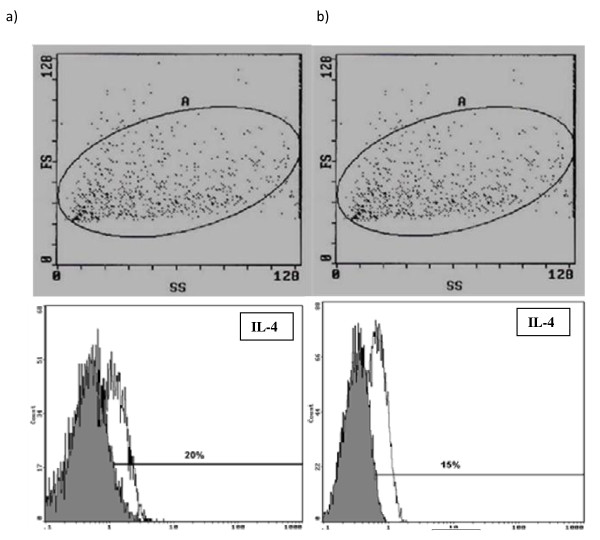
**Identification of IL-4 in thyroid follicular cells in patients with GD and HT using flow cytometry**. (a) Flow cytometric dot plots of dispersed TFCs from a representative patient with GD. Scatter plot of gating thyrocytes (left upper panel). Flow cytometric detection of IL-4 in thyrocytes from patients with GD (left down panel). Cells were labeled with control IgG1 (grey) or anti-IL-4 mAb (white). (b) Flow cytometric dot plots of dispersed TFCs from a representative patient with HT. Scatter plot of gating thyrocytes (right upper panel). Flow cytometric detection of IL-4 in thyrocytes from patients with HT (right down panel). Cells were labeled with control IgG1 (grey) or anti-IL-4 mAb (white).

**Figure 3:**
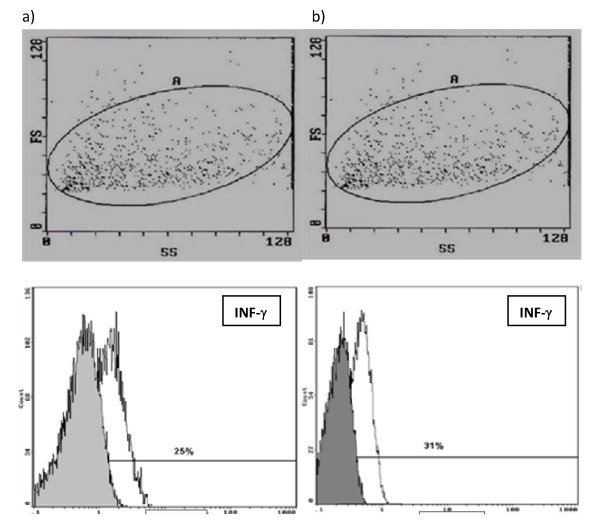
**Identification of INF-γ in thyroid follicular cells in patients with GD and HT using flow cytometry**. (a) Flow cytometric dot plots of dispersed TFCs from a representative patient with GD. Scatter plot of gating thyrocytes (left upper panel). Flow cytometric detection of INF-γ in thyrocytes from patients with GD (left down panel). Cells were labeled with control IgG1 (grey) or anti-INF-γ mAb (white). (b) Flow cytometric dot plots of dispersed TFCs from a representative patient with HT. Scatter plot of gating thyrocytes (right upper panel). Flow cytometric detection of INF-γ in thyrocytes from patients with HT (right down panel). Cells were labeled with control IgG1 (grey) or anti-INF-γ mAb (white).

Fresh thyroid tissues were taken on culture medium RPMI -1640, it was mechanically preparated (described as above). Briefly, the cell suspensions were rinsed three times with PBS, and later were added cell activators - 12 - myristate 13 - the acetate (the PMA - 50 ng/ml) and Ionomycin (1 mM) as well as the inhibitor of transportation of proteins - Breferdin A (3 mmol/ml). They were cultured 24 hours in 50 ml flasks at 37°C in a 5-95% CO2-air water-saturated atmosphere. After that, 10 μl of mouse monoclonal antibody (mAb) directed against human TPO epitope 64 was added into the suspension. After 15 minutes of incubation at room temperature to each of samples added 100 μl of reagent A fixative the cellular membrane (IntraStain-Dako, Denmark). In next step we conjugated this complex with rabbit anti-mouse antibodies IgG (Fab')_2 _labeled by FITC and than incubated by next 15 minutes (identified as above). Then, the cell suspensions were rinsed three times with PBS and were added 100 μl of reagent B to permeabilization the cellular membrane (IntraStain-Dako, Denmark) and 20 μl specific anti-IL-4-PE (IgG_1_, BD) or anti-IFN-γ-PE (IgG_1_, BD) monoclonal antibody, and murine IgG_1 _as negative control, then incubated for 30 min at 4°C. After two washes with PBS supplemented with 5% BSA, the cells were immediately analyzed in a flow cytometry (Coulter EPICS XL) (Figure 2, Figure 3).

### Identification of intracellular IL-4 and IFN-γ levels in lymphocyte populations by flow cytometry

The percentage of lymphocytes was determined as previously described [8]. Briefly, after 24 h of incubation with cell activators and inhibitor of transportation of proteins (described above), the non-adherent cells (predominantly lymphoid cells and erythrocytes) were removed from culture supernatants for lymphocyte isolation. In the next step, 10 μl anti-CD4-PerCP and anti-CD8-PerCP (IgG1, BD) monoclonal Abs were used for direct staining of membrane molecules specific for T lymphocytes. After 15 minutes of incubation at room temperature to each of samples added 100 μl of reagent A fixative the cellular membrane (IntraStain-Dako, Denmark) and incubated by next 15 minutes. Then, the cell suspensions were rinsed three times with PBS and were added 100 μl of reagent B to permeabilization the cellular membrane (IntraStain-Dako, Denmark) and 20 μl specific anti-IL-4-PE (IgG_1_, BD) or anti-IFN-γ-PE (IgG_1_, BD) monoclonal antibody, with murine IgG_1 _as negative control. The cells were analyzed in a flow cytometry (Coulter EPICS XL).

### Statistical analysis

The results were analyzed using Statistica 9.0 software. The mean values of immune parameters between groups were evaluated using Student's t test, U Mann-Whitney's test or Fisher's exact probability test. Correlation was assessed by Spearman's signed ranks test. P < 0.05 was considered significant.

## Results

Table 1 presents the characteristics and laboratory findings in patients with GD (prior to methimazole treatment and then in the state of clinico-biological euthyrosis before surgery), with HT and with nontoxic nodular goiter (NTMG; prior to treatment). Patients with > 1 cm nodular lesions in ultrasonography underwent fine-needle aspiration biopsy (BAC), which showed benign colloid nodular goiter. Scintigraphy additionally performed in 3 patients with nodular goiter and clinical symptoms of hyperthyroidism showed a selective increase in the accumulation of radioactive iodine within the area of single nodular lesions.

**Table 1:** Data of patients with GD before methimazole therapy, after six months of therapy (prior to surgery) in non-toxic multinodular goiter and HT

	Untreated GD group Group A	Group after six months of methimazole therapy Group A'	Group with HT Group B	Group with non-toxic multinodular goiter Group C	P
**Age (years)**	15.4 ± 2.1	16.4 ± 3.3	16.8 ± 2.8	15.8 ± 1.8	NS, NS*, NS**

**n**	18	18	18	18	

**Weight (kg)**	57 ± 11.0	59 ± 7	63 ± 8	62 ± 7	NS, NS*, NS**

**Height (cm)**	166 ± 3	169 ± 1	170 ± 5	169 ± 7	NS, NS*, NS**

**BSA (m^2^)**	1.66	1.68	1.7	1.67	NS, NS*, NS**

**anti-TPO (IU/ml)**	1250 ± 350	650 ± 250	1550 ± 500	41.3 ± 38	p < 0.0001, p* < 0.01, p** < 0.001

**anti-TG (IU/ml)**	630 ± 262	360 ± 40	820 ± 150	180 ± 86	p < 0.001, p* < 0.01, p** < 0.001

**TRAb (U/l)**	> 5 positive	> 3 positive	(-)	(-)	

**fT4 (ng/dl)**	5.9 ± 2.0	2.04 ± 0.3	1.29 ± 0.2	1.1 ± 0.2	p < 0.001, NS*, NS**

**fT3 (ng/dl)**	8.85 ± 2.1	3.8 ± 0.5	3.64 ± 0.21	2.94 ± 0.21	p < 0.002, NS*, NS**

**TSH** **(μIU/ml)**	0.03 ± 0.01	1.3 ± 0.2	3.1 ± 0.5	2.9 ± 0.3	p < 0.001 p* < 0.01, NS**

**GD- **Graves' disease **HT- **Hashimoto's thyroiditis **NTMG-**non-toxic multinodular goiter

p, Statistical significance between group A and C; p*, statistical significance between group A' and C; p**, statistical significance between group B and C;

X, standard deviation; NS, no statistical significance.

In the analysis of the thyroid peroxidase antigen by flow cytometry method, anti-human native TPO mouse monoclonal antibodies were used. The assessment of the percentage of thyroid cells with mAb #64TPO epitope expression showed no significant differences between GD and HT patients (73 vs.65%, NS) at a concentration of 400 mg/ml of anti-TPO antibodies. When the concentration of anti-TPO antibodies decreased, the mean percentage of thyroid cells with antigen region #64 expression was reduced.

**Figure 4:**
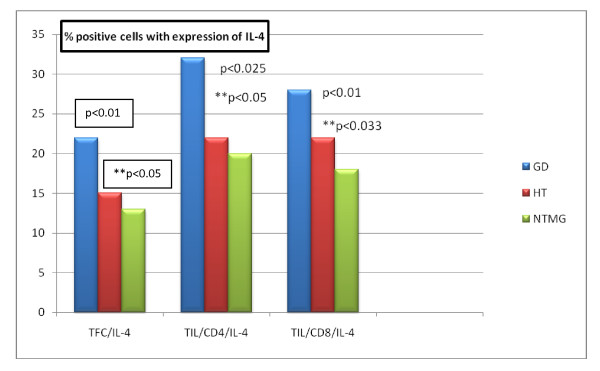
**Mean percentages of positive TFCs and TILs with intracellular expression of IL-4 in examined patients**.

Patients with GD showed a higher percentage of CD4+IL-4, CD8+IL-4 and TFC-IL-4+ cells, compared to patients with HT (p < 0.05, p < 0.033, p < 0.05, respectively) and NTMG (controls) (p < 0.025, p < 0.01, p < 0.01, respectively) (Figure 4). There was no difference between the proportions of these CD4+IL-4, CD8+IL-4 and TFC-IL-4+ cell populations in cases with HT and NTMG.

**Figure 5:**
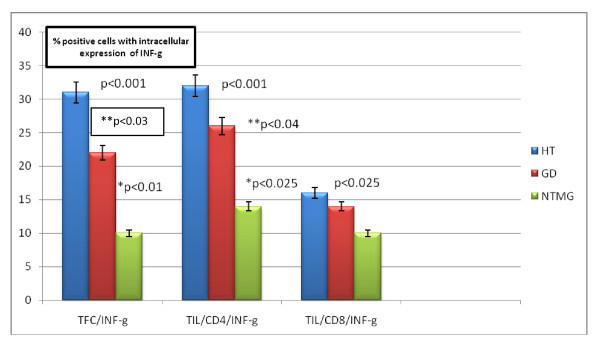
**Mean percentages of positive TFCs and TILs with intracellular expression of INF-g in examined patients**.

The analysis of mean percentages of positive TILs and TFCs with intracellular INF-g levels in patients with HT revealed statistically significant increase percentage of CD4+INF-γ, CD8+ INF-γ, TFC+INF-γ cells, compared to the percentage of positive cells from patients with GD (p < 0. 04, NS, p < 0.03, respectively) and NTMG (p < 0.001, p < 0.025, p < 0.001, respectively) (Figure 5).

In the present study, the relationship between antithyroid antibodies and the percentage of TFCs and T cells with the expression of cytokines was evaluated. In patients with untreated GD there was a statistically significant positive correlation between the percentage of TFCs-IL4+ and CD4+IL-4 T cells and the concentration of TRAK antibodies (R = 0.62, p < 0.02; R = 0.34, p < 0.04, respectively). However, no such correlations were noted in regard to patients with HT and non-toxic multinodular goiter. There were no significant correlations between thyroid hormones and the percentage of intrathyroid lymphocytes and TFCs with IL-4 and INF-γ expression.

## Discussion

An altered balance of pro- and anti-inflammatory cytokines is thought to play an important role in the pathogenesis of autoimmune thyroid diseases. Disruption of thyroid self tolerance, usually after an infection, generates abnormal thyroid-immune interactions, implicating an array of cytokines and their receptors. Thyrocytes achieve antigen presenting cell properties, which stimulate effectors immune cells: Th1 and Th2, in the context of defective immunomodulatory T regulatory cells, resulting in thyroid lymphocytic infiltration and activation of B cells, with production of antibodies against thyroid antigens, thyroid destruction or stimulation, depending on the Th1-Th2 balance [9].

Over the last decade many researchers have proven, that T cell recognition of autoantigens had shown considerable intra- and interindividual heterogeneity, and a mixed pattern of cytokine production indicates that both the Th1 and Th2 limbs of the helper T cell response are involved in all types of autoimmune thyroiditis. HT glands activate T cells that display a predominantly Th1 phenotype. The Th1 pattern produces IL-2, IFN-γ, and TNF-α, with a relative absence of IL-4 or -10, suggesting that these cells support a predominantly T cell-mediated induction of apoptosis [10]. In contrast, GD thyrocytes do not undergo apoptosis and are characterized by a predominantly Th2 infiltrate [11]. The Th2 pattern produces relatively less IL-2, TNF- α, and IFN- γ, and more IL-4, -5, and -10, indicating a tendency toward B-cell maturation and an antibody-mediated immune response [10].

Secretion of cytokines within the thyroid accounts for the accumulation and expansion of the intrathyroidal lymphocyte pool. Also, the thyroid cells themselves contribute to this secretion. The thyroid cells also produce a number of proinflammatory molecules which will tend to exacerbate the autoimmune process, eg. IFN- γ and IL-4 [12].

IFN- γ, a prototypic proinflammatory cytokine produced by several different cell types, including the Th1 subset of CD4(+) T cells, plays an important role in inflammation and autoimmune diseases [13]. Carella C et al. observed that INF- γ is the cytokine most clearly associated with AITD, especially hypothyroidism, despite being neither Th1- nor Th2-dependent [14]. Phenekos C et al. found that patients with HT had higher INF- γ levels compared to patients with TNG, GD and controls. In contrast, patients with GD had higher serum levels of IL-4 in comparison with patients with HT. These results were similar to the results of our study. Therefore, we can also conclude, that a Th1 pattern of immune response characteristic of cellular immunity is dominant in HT, whereas the predominance of Th2 cytokines in GD indicates a humoral pattern of immune reaction [15].

IL-4 is produced by Th2, mastocytes and basophiles. The IL-4 is involved in both humoral and cellular immunity. Chen RH et al. tested whether the IL-4 gene could be used as a genetic marker to predict the development of AITD among the Chinese population of Taiwan. They found no significant difference in the frequencies of presence of genotype and allelic variants for the IL-4 gene at both the intron 3 and the promoter regions between the normal control group and each of the two patient groups. These findings suggest that the IL-4 gene polymorphisms that arise at either intron 3 or promoter -590 positions are not suitable genetic markers for AITD among Taiwanese Chinese [16].

Because GD and HT are a common autoimmune disorders with genetic predisposition, the expression of genes coding cytokines was measured. Zhu W et al. observed strong evidence that the Chr.5q31-33 region, which contains the immune response various cytokine genes eg. IL-4, is linked to autoimmune thyroid disorders in Chinese and Japanese populations [17]. Also Khalilzadeh O et al. observed the association between GD and the polymorphisms in anti-inflammatory cytokine IL-4 [18].

Alnaqdy A et al. noticed that patients with GD showed significantly higher levels of IL-4 and IFN- γ compared with the normal controls. There was also a significant increase in serum levels of IL-4 in the treated group, compared to the untreated group. IL-4, and IFN-γ, showed a high prevalence in Omani patients with GD. Thus, cytokines and autoantibodies may prove useful in the diagnosis of GD and in assessing prognosis [19].

Worth emphasizing is fact, that IL- 4 in GD prevents adhesion molecules expression in order not to damage thyrocytes. In our study production of IL-4 in thyrocytes of patients with GD was elevated, what may be responsible for protection of these cells against immune damage or reduction of their apoptosis. Stassi G et al. suggested that, infiltration of Th2 lymphocytes and production of IL-4 and IL-10 by thyrocytes in patients with GD can induce the expression of antiapoptotic molecules and leading thyrocyte resistance to CD95-mediated apoptosis [20]. On the other hand, in patients with HT we detected in thyrocytes elevated level of IFN- γ, what may be responsible for prolongation of inflammatory process within thyroid gland.

In conclusion, our findings suggest that the human thyrocytes in autoimmune thyroid disorders could be a source of cytokine production and that their activation influences local interaction with T lymphocytes inflowing. The alteration of intracellular cytokine levels in T cells within thyroid gland suggest predominance role of Th1 immune response in HT, and development of Th2 immune response in GD.

## Abbreviations

**PerCP**: peridinin chlorophyll protein; **FITC**: fluorescein isothiocyanate; **PE**: phycoerythrin; **fT3**: free triiodothyronine; **fT4**: free thyroxine; **TSH**: thyroid stimulating hormone; **TRAbs**: antibodies against receptor for thyroid stimulating hormone; **TPO-Abs**: antithyroid peroxidase antibodies; **hTPO**: human thyroid peroxidase; **TG-Abs**: antithyroglobulin antibodies; **AITD**: autoimmune thyroid disease; **GD**: Graves' disease; **NTMG**: non-toxic multinodular goiter; **HT**: Hashimoto's thyroiditis; **mAb**: monoclonal antibodies; **TFC**: thyroid follicular cell; **ITLs**: intrathyroidal T lymphocytes.

## Competing interests

The authors declare that they have no competing interests.

## Authors' contributions

AB(1) participated in the designing of the study, collecting of clinical data and drafting the manuscript. MH and AB(4) performed the statistical analysis and helped to draft the manuscript. JH performed thyroid surgery and collected the clinical material. KR participated in the cytometric analysis. AM participated in the design of the study and drafted the manuscript. All authors read and approved the final manuscript.
